# Variable sexually dimorphic gene expression in laboratory strains of *Drosophila melanogaster*

**DOI:** 10.1186/1471-2164-8-454

**Published:** 2007-12-10

**Authors:** Dean A Baker, Lisa A Meadows, Jing Wang, Julian AT Dow, Steven Russell

**Affiliations:** 1 Department of Genetics, University of Cambridge, Downing Street, Cambridge CB1 3QA, UK; 2 Cambridge Systems Biology Centre, Tennis Court Road, Cambridge CB2 1QR, UK; 3 Division of Molecular Genetics, Institute of Biomedical and Life Sciences, University of Glasgow, Glasgow G11 6NU, UK

## Abstract

**Background:**

Wild-type laboratory strains of model organisms are typically kept in isolation for many years, with the action of genetic drift and selection on mutational variation causing lineages to diverge with time. Natural populations from which such strains are established, show that gender-specific interactions in particular drive many aspects of sequence level and transcriptional level variation. Here, our goal was to identify genes that display transcriptional variation between laboratory strains of *Drosophila melanogaster*, and to explore evidence of gender-biased interactions underlying that variability.

**Results:**

Transcriptional variation among the laboratory genotypes studied occurs more frequently in males than in females. Qualitative differences are also apparent to suggest that genes within particular functional classes disproportionately display variation in gene expression. Our analysis indicates that genes with reproductive functions are most often divergent between genotypes in both sexes, however a large proportion of female variation can also be attributed to genes without expression in the ovaries.

**Conclusion:**

The present study clearly shows that transcriptional variation between common laboratory strains of *Drosophila *can differ dramatically due to sexual dimorphism. Much of this variation reflects sex-specific challenges associated with divergent physiological trade-offs, morphology and regulatory pathways operating within males and females.

## Background

An important proportion of phenotypic evolution and variation is the result of changes in gene expression. In order to investigate the molecular processes that lead to divergence in transcript levels between strains or even related species, it is necessary to partition environmental effects of variation from other genetic components. Towards these ends, the use of gene expression estimates derived from microarray experiments have become an increasingly popular tool for attempting to close the gap between genotype and phenotype [1-4]. Results from such studies suggest that, unlike phenotype, the heritable components of gene expression are largely non-additive with epistatic and genotype-environment interactions playing a far greater role in controlling transcriptional abundance than previously expected [5-7]. Such findings have important implications for understanding the maintenance and divergence of transcription levels. Here, we investigate variance observed between males and females of *Drosophila melanogaster *laboratory strains at the gene expression level. Many inbred lines, including wild-type stocks of *Drosophila*, have typically been generated from populations with segregating genetic backgrounds that contain multiple mutations. Our analysis is not designed to make inferences about evolutionary history or heritability, rather it aims to describe the extent to which significant transcriptional variation is inherent in commonly used laboratory strains.

In particular, we wished to determine whether specific classes of gene show variation in transcriptional abundance and whether these relationships are related to sexual dimorphism. It has been reported previously that both the sex and tissue in which a gene is expressed acts to influence the degree of sequence level variation apparent in populations [8-10]. For example, *Drosophila *sequence variation in male reproductive genes is over-represented and appears to be consistently evolving under positive selection [11-14]. By comparison, although some female reproductive genes evolve under positive selection, many do not evolve as quickly as male reproductive genes, and it has been suggested that they are also influenced by balancing selection [15-17]. The accumulation of mutations associated with different aspects of reproduction may subsequently be over-represented and influence transcriptional variation of males more than females. In addition, as laboratory strains are typically kept in isolation for many years, if not decades, strains continue to accumulate transcriptional differences that reflect mutation, genetic drift and selection (or lack there of). Inbreeding depression and the accumulation of deleterious mutations in this fashion is known to have a significant impact on gene expression [18,19].

Ultimately however, variation in gene expression is very likely to be influenced by multiple underlying mechanisms. On the one hand, mutations in regulatory sequences may result in transcriptional differences between strains, but it is also probable that variation reflects the sensitivity of any particular process to the accumulated effects of transcriptional variation. Male fertility and spermatogenesis has received particular attention in this regard. Investigation of post-zygotic reproductive isolation of hybrids has lead to the suggestion that male-biased genes with reproductive functions are more easily perturbed than female-biased genes [20-23]. Indeed at the transcriptional level, male-biased genes display considerable variation both within and between species, while female-biased gene expression appears to have a reduction in the amount of variation apparent in populations [24]. In terms of female-biased variability however, it is important to note that these relationships have not been confirmed through direct investigation of variation in female transcription between genotypes, something we aim to address in the current study.

## Results and Discussion

Our goal was to identify genes that display transcriptional variation between strains of *Drosophila *and to explore evidence of gender-biased interactions underlying that variability. Towards this end, we performed whole-genome microarray analysis, examining relative expression levels in adult males and females that: 1) were variable between genotypes, 2) were variable specifically in each sex, and 3) showed either reproductive tissue or somatic expression. For the latter analysis, we integrated data with a recently generated tissue-specific dataset from FlyAtlas [25].

After normalization (see methods), differential expression for Sex, Genotype and each Sex × Genotype interaction was investigated with a linear model comparing these contrasts of interest. Linear models are an increasingly popular method for exploring microarray data, where an analysis of variance is fit to each probe and a formal statistical threshold applied to obtain lists of differentially expressed genes. Confidence in differential expression measures can then be improved by utilising a pooled estimate of sample variance with empirical Bayes due to the parallel nature of microarray datasets [26]. The statistical significance for each of the biological effects after Bonferroni correction are given in Table 1.

**Table 1:** Significance level of experimental treatments after Bonferroni correction for each contrast of interest.

**Interactions**	**Significance level**			
	***0.0001***	***0.001***	***0.01***	***0.05***

Sex	5490	6020	6503	6876
Genotype	637	1041	1720	2920
Female × Genotype	334	570	920	1280
Male × Genotype	449	719	1157	1753

In *Drosophila*, it has been widely reported from microarray studies that the majority of transcriptional variation identified within strains is due to gender differentiation with as much as 50% of the genome showing variable gene expression [1,27]. We find that, depending on the statistical threshold, 39% to 49% of variability can be attributed to sexual dimorphism (Table 1). This degree of variation, as well as the chromosomal locations of probes with differential expression (Data Not Shown; Fishers Exact Test; Bonferroni Correction; p < 0.05) is in agreement with previous studies showing an enrichment of female-biased and a paucity of male-biased expression on the *X *chromosome in contrast to the autosomes [2]. As was expected, genotypic expression averaged across gender shows a lower degree of variation than male versus female expression averaged across genotype (Table 1). The number of probes showing variability between at least one pair-wise interaction ranges between 4% and 20% of probe sets, depending on the statistical threshold applied. These figures are within the range of earlier studies contrasting gene expression between laboratory strains [1,5].

The contrasts of the linear model were also analysed to establish male and female specific interactions between genotypes (Table 1). In order to develop a high confidence list of genes variable in either males or females, we filtered each gender interaction with even greater control on variable expression (see methods). Normalized intensity values and F-statistics for each interaction are provided in Additional File 1 and 2. The resulting set of genes show variability between genotypes in a single sex, but are not necessarily gender-biased in expression. In order to then determine whether enrichment for male or female-biased gene expression is apparent in these interactions, genes were selected with a greater than 2-fold difference in expression level between sexes and with a formal statistical threshold (p < 0.01). The expected proportions for male-biased, female-biased and unbiased expression in the dataset have been provided with the use of enrichment statistics (Table 2).

**Table 2:** Whole tissue expression categories in Sex × Genotype Interactions. Significance indicated in bold (over-representation) or *italics *(under-representation) text for a Two-tailed Fishers Exact Test (p < 0.05). Numbers in parentheses indicate the number of genes in each category. Numbers enclosed by square brackets indicates the number of genes with single sex expression.

	Male-Biased (1835)	Female-Biased (2385)	Unbiased (4161)
**Male × Genotype (563)**	**202 **[150]	***41***	321
**Female × Genotype (481)**	**159**	140 [68]	***182***

Transcriptional variation among genotypes occurs more frequently in males than variation in females (Table 2). After discarding overlap within each dataset, of the remaining 563 Male × Genotype genes, 36% were male-biased in expression, a proportion significantly greater than expected by chance given the number of male-biased genes on the whole array (Fishers Exact Test; p < 0.05). Female-biased expression by comparison was under-represented in the male interaction dataset occurring in only 7% of genes (Fishers Exact Test; p < 0.05). In female interactions, an over-representation of genes with gender-biased expression was also found. Of the 481 Female × Genotype genes, 33% were male-biased in expression (Fishers Exact Test; p < 0.05). In the case of female-biased expression however, while 29% show high rates of transcription in females, this total is not enriched and is expected to occur by chance given the number of female-biased genes from whole genome comparisons. Genes that were unbiased in expression were further identified as under-represented within female interactions. A moderate proportion of genes were found to be expressed in only one gender (Table 2). In female interactions, 68 genes were expressed only in females, while in male interactions, 150 genes were found to be expressed only in males.

We next looked to determine whether variable genes within each gender interaction were related by functional annotation. Using several high-level Gene Ontology (GO) terms we partitioned each gene list into 14 categories (Table 3). Male and female interactions typically show a limited amount of overlapping function. Both gender interactions are, for example, over-represented for genes involved in the defence response, metabolism and transport. Over-representation of functional classes unique to either male or female interactions were, however, also apparent. Male specific over-representation is found for mitochondrial function, whereas female specific over-representation relates to genes with developmental, proteolytic or signal transduction functions. It is important to note that male and female interactions primarily display a different set of genes even in the same functional classes. As expected, the number of genes with unknown function was high.

**Table 3:** Genes with GO Annotation in each Sex × Genotype interaction. Gene enrichment is shown in bold at a significance level of p < 0.05.

**Annotation Category**	**Male**	**Female**
Behaviour	16	11
Cell Cycle	21	27
Defence Response	**29**	**34**
Development	56	**87**
Metabolism	**273**	**192**
Mitochondrial	**39**	3
Proteolysis	34	**25**
Response to Toxin	7	8
Signal Transduction	59	**66**
Structural	32	31
Synaptic Transmission	11	13
Transcription	17	34
Translation	19	14
Transport	**110**	**81**
Unknown	203	178
Total No. of Genes	563	481

Between genotypes, variation in genes associated with innate immunity is higher than expected by chance. Several anti-microbial mechanisms exist in *Drosophila *that have distinct target specificities and are controlled by alternate signalling cascades, of which the anti-fungal and antibacterial responses of the Toll and Imd pathways are known in particular detail [28]. The Toll pathway is primarily responsible for control of the anti-fungal response mediated by *drosomycin*, while expression of anti-bacterial peptides like *defensin*, which we find to be variable between genotypes regardless of sex, requires input from both pathways. As genotypes were kept under the same laboratory conditions, infection pressure is not expected to differ between the strains. However, is it possible that some genotypes are more susceptible to infection than others, thus contributing to observed variation in anti-bacterial response genes.

Analysis of the relationship between inbreeding depression and transcription have revealed that genes associated with metabolism and defence response are disproportionately influenced by the method of establishing genotypes [18,19]. Slower rates of inbreeding for example are expected to be less deleterious than faster inbreeding rates, allowing greater opportunity for selection to act before a given level of genetic diversity is lost. The genotypes included in this current study were established at different times, from different locations and presumably by different methods. As a result, we expect that the segregation of transcriptional diversity is strongly linked to effective population size regardless of the ultimate combination of inbreeding depression, founder effects and genetic drift responsible. Clearly, the accumulation of mutations will also lend to inter-genotype variation, and our strains have been in the laboratory for different periods of time. Rifkin et al have shown that in as little as 200 generations, mutational variation can appear in up to 39% of the genome, a factor we expect to contribute to the transcriptional variation we observe [29].

While we find that male and female interactions are variable for genes associated with the immune response, it is widely recognized that the majority of sex-biased regulation in *Drosophila *is the result of expression in germ-line tissues [2,30]. Similarly in our data, the majority of variable genes between genotypes in males or in females are indeed expressed in the reproductive system. Tissue specific microarray data for the testis and the ovaries were taken from the FlyAtlas database [25], and integrated with male and female interaction lists to identify genes with expression in reproductive tissue or with expression restricted to somatic tissues (Table 4). It is readily apparent that genes from both male and female interactions frequently occur in the reproductive tissue, while genes with restricted somatic expression occur less frequently.

**Table 4:** Presence calls of Testis, Ovary and Somatic Gene Expression.

**Male × Genotype**	Male-Biased	Female-Biased	Unbiased
Testis	153	38	236
Somatic Only	49	3	85
			
**Female × Genotype**			
Ovary	80	129	151
Somatic Only	78	11	30

In invertebrates, reproductive tissues often contain a high number of immunity related molecules. Males in particular express a range of anti-microbial peptides in the reproductive tract including the male accessory glands [31]. Immunity molecules in the reproductive tissue of females are typically associated with the oviduct and sperm storage [32]. The relationship between reproduction and immunity is emerging as very complex, in which genetic trade-offs act to balance reproductive investment against fitness. This suggests that sexual selection is an important evolutionary mechanism acting to drive increased mating effort to the detriment of mounting defence responses [33,34]. Moreover, immunity-related genes are often driven by positive or balancing selection at the sequence level, showing the importance of adaptation on such traits and indicating that the segregation of divergent alleles in laboratory strains also represents a potential source of the variance detected [35-38].

An important component of cellular defence is the expression and regulation of proteolytic enzymes [39], and we find that variability in the expression of genes encoding this function is over-represented (Table 3). Included in this list are components of the Toll pathway and the immune response of flies (*snk*, *psh*) [40], but in addition three protease regulators linked specifically with reproduction and the male accessory gland are present (*Acp76A*, *Spn2*, *Spn3*). The male accessory gland is responsible for producing and secreting a mixture of proteins, the male accessory gland proteins (Acps), forming seminal fluid that is transferred along with sperm to females during copulation [41]. Many Acps are believed to be activated in females after mating by proteolytic activity, whereas in males protease inhibitors are thought to keep proteases inactive until they reach their destination in the spermathecum. One third of Acps are passed on to females during mating, including some serpins [42,43], and in addition to the three protease regulators mentioned, at least two more Acps in our dataset are variable in males between genotype (*lectin-28C*, *lectin-46Cb*).

Molecules from the male accessory gland have received considerable attention in the literature because, when passed to females during mating, they induce a variety of changes in gene expression. In cases for which functional data on the action of Acps exists, female targets include not only genes expressed specifically in the ovaries, but also genes expressed in female somatic tissues, including the spermatheca, uterus and even the hemolymph. Gene expression changes in these target tissues are associated with female receptivity, ovulation, egg production, defence response and longevity [41-43]. Very few examples from our female variable dataset correspond to genes whose regulation is known to be induced by mating [44,45]. One of the few exceptions however is, *upheld*, a Troponin that forms part of the Troponin complex associated with actin in muscle thin filaments as well as calcium regulation of muscle contraction [46]. Changes in muscle contraction are likely to underlie some effects of Acp regulation related to ovulation, egg deposition, or sperm storage. When all levels of the GO hierarchy were subsequently considered, the highest significance levels indicate that several genes linked to muscle contraction are over-represented in the female interaction dataset (*Mp20*, *sls*, *Tm2*, *CaMKII*, *Mbs*, *bt*, *GstS1*, *TpnC73F*, *Rya-r44F*, *CG1776*, *CG5023*, *zormin*).

**Figure 1:**
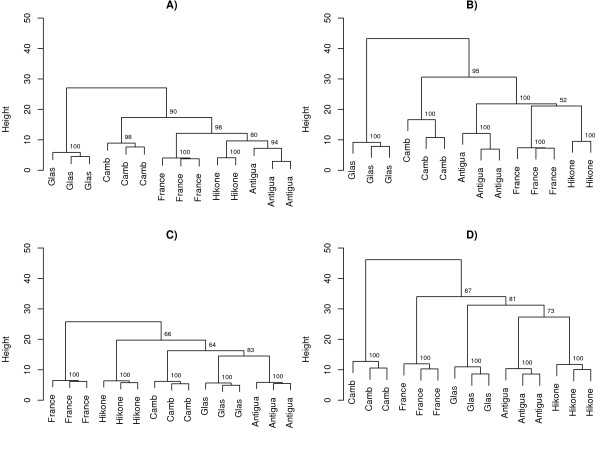
Visual representation of genotypic distance in tissue and gender gene expression datasets. a) Female Interactions; Somatic Tissue b) Female Interactions; Reproductive Tissue, c) Male Interactions; Somatic Tissue, d) Male Interactions; Reproductive Tissue. Clustering was achieved with euclidean distance on centred gene expression data. Numbers at each node of a tree indicate the bootstrap confidence after 1000 replications.

While there is then evidence from functional annotation and spatial expression to suggest that male and female variation occurs primarily in different sets of genes, it is unclear how each genotype contributes to the overall variation we observe and whether these effects are correlated. We have subsequently clustered genotypes for each gender interaction and tissue combination in an effort to determine whether global variation in gene expression displays similar patterns between treatments (Figure 1). Hierarchical clustering was performed on male and female interaction sets by partitioning each into genes with expression either present or absent from the testis in male interactions, or the ovaries in female interactions. Support for branches was estimated with bootstrap analysis (n = 1000).

The goal of clustering is to make statistical inference about discrete structures, and high bootstrap values at each node indicate that a tree is particularly robust. Here, tree topology is based on transcription. Branch lengths in females (Figure 1a,b) and males (Figure 1c,d) reflect the distribution of variation recorded within and between strains. As expected, replicates cluster with short branch lengths and strong bootstrap support in both sexes and in both tissue comparisons at the tree edges. In female interactions we also find that there is generally good support for the clustering of internal branches. Genotypes from female interactions tend to display the same distance rankings regardless of the tissue in which genes are expressed. In particular, we find that the Glasgow and Cambridge genotypes are consistently the most distant in both ovary and somatic tissues. By comparison, male interactions display variation in the ordering of genotypes from testes and somatic tissues, especially in regard to those which are the most distant. While it is important to note that bootstrap support for internal branches in male interactions shows the presence of further uncertainty, these findings suggest that different mechanisms of variation are possibly acting on genes within the reproductive and somatic tissue of male interactions, but not in female interactions.

In general, functional constraint on gene expression in male reproductive tissues is known to be lower than for other genes, which is reflected in the coupling of expression variation with faster rates of sequence evolution [9,16]. Indeed, sequence level analysis of male reproductive genes consistently shows that they are evolving under positive selection with divergence apparent between populations [10,14,24]. The lack of functional constraint in male-biased transcriptional variation is also apparent in studies of hybrid incompatibility, which suggest that mis-regulation of gender-biased genes is a common occurrence in males and an important mechanism for reproductive isolation [47]. Hybrid males of closely related *Drosophila *species are normally infertile, and genes expressed in the testis that have roles in sperm differentiation and maturation show particularly high levels of expression incompatibility [27,48-50].

Our dataset indicates that genes from male interactions are predominantly found in the testis, suggesting a strong link with reproductive function (Table 4). To confirm this hypothesis we further annotated our gene set with additional public expression data, using a list of testis-specific genes from tissue-specific microarray data along with EST data from testis, head and ovary cDNA libraries [51]. Testis-specific expression was confirmed for 61 genes and these were found to be distributed in a gene cluster that is dominated by male-biased gene expression as shows in Additional File 3. GO annotation also supports the view that many male variable genes are linked to testis functions, since we observe enrichment for GO terms associated with energy production. This is consistent with the expectation that sperm production is reliant on mitochondrial activity and metabolic functions. Furthermore, we find several genes are directly annotated as having roles in spermatogenesis (*Act5c*, *bel*, *crl*, *fbl*, *lectin-28c*). Such findings are in accord with higher rates of mutation and the disruption of genes with roles in sperm development, which are likely to be under particularly high rates of sequence and transcriptional evolution [52,53].

While female interactions are also often dominated by genes expressed in the ovary, very few genes are known to be ovary specific in our investigation of EST expression. However, in females the early stages of egg development form part of regulatory cascades that direct later embryonic development [54]. Sequence level studies have shown that while female reproductive genes can show evidence of evolutionary pressure, the type and strength of selection is more variable than typically found in males, *i.e. *both positive and balancing selection [8,10]. Such findings suggest that many components of egg production are well conserved. Yet during ovarian follicle cell development, at least four genomic intervals are over-replicated in a modification of the cell cycle, increasing the amount of DNA template available for transcription [55]. High levels of mRNAs are necessary for the formation of chorion layers surrounding the oocyte, with female sterility and gross abnormalities of chorion structures resulting when genes influencing gene-amplification are mutated [56,57].

The process of gene-amplification involves initiation of DNA replication at specific points in the genome, facilitated by a complex of proteins which recruit the machinery necessary for DNA synthesis [58]. While targets of amplification exhibit a 4- to 80-fold increase in copy number [55], a decreasing and variable gradient in copy number extends approximately 50 kb either side of the central target genes [59]. Our female variable dataset contains genes from three of the four known chromosomal target regions implicated in ovarian gene-amplification and the 50 kb flanking regions. The chromosomal regions, 7F on chromosome *X *and 30B on chromosome *2L*, are each represented by a single gene, *sprite *and *Gdi *respectively, in the core amplification regions. Similarly, the 62D region on chromosome *3L *contains two genes that are variable in females between genotypes, *oxt *and *yellow-g2. *While *yellow-g2 *and *oxt *products are thought to be involved in the formation of the eggshell and/or crosslinking of the chorion [55], the role of *sprite *and *Gdi *in eggshell formation is unclear.

Although we then find there is particularly high divergence of genes with reproductive functions, and the distance recorded between genotypes is greater in reproductive tissue (Figure 1b,d) than in somatic tissue (Figure 1a,c), an interesting observation in female variable genes with restricted expression to the somatic cells, is that at least half of these genes are highly expressed in males from whole-tissue comparisons (Table 4). In particular, many of these genes are related to signal transduction, and components of sensory reception. Several genes were identified with roles in phototransduction (*ninaE*, *ogre*, *Rh3*, *Rh4*). Other members of this group however are known to participate in both visual and olfactory neurotransmission, such as the *arrestins *(*Arr1*, *Arr2*), mediators of neurotransmission through G-protein coupled receptor cascades [60], and *trp *whose functions are integral for visual and olfactory adaptation [61]. While male flies have fewer ommatida than females, visual-input is an important mediator of behaviour and there is evidence of sexual dimorphism in the structure and function of dipteran eyes [62,63].

Somatic genes with a requirement for high levels of gene expression in males, but not in females, may be strongly influenced by the sex-determination pathway. Gender-specific patterns of growth, morphogenesis and differentiation are controlled by the action of several well known factors including *Sex lethal*, *doublesex*, *transformer *and *fruitless *[64]. Genomic investigation of mutations in the sex-determination pathway have found that approximately 1.5% of genes are regulated in the soma downstream of *transformer *by either Fruitless or Doublesex [3]. Several such genes overlap with our female variable dataset, two of which are linked to mutations of *doublesex *(*Rh4*, *Nrx-1*). While the sex-determination pathway in *Drosophila *is well understood, the genes which are regulated by the hierarchy have only begun to be identified. Further examination of such genes is likely to provide important insight into transcriptional variation and its relationship to sexual dimorphism.

## Conclusion

The large number of differentially expressed genes we found in this study has excluded the possibility of presenting any detailed gene-wise description of the variation contained therein. Instead we primarily present trends in the form of genes that can be grouped together by broader function. Although many of the functional categories that vary between the genotypes we investigated are common to males and females, overlap in the actual genes presented are generally limited at this level of analysis, and unique components of genetic variation can be attributed to one gender or the other. In both sexes, for example, genes involved in the defence response, transport and metabolism are disproportionately affected by variation in gene expression between genotypes. Yet, many of these interactions are variable for different genes in each gender. Such sexual dimorphism most likely reflects challenges that are unique to males or females in the context of morphological differences, physiological trade-offs and differential gene regulation between genotypes.

The purpose of this investigation was to investigate the nature of Sex × Genotype variation between strains of long-term laboratory maintained stocks. In such a scenario many of the genes found to vary between genotypes are likely to reflect differences associated not only with the effects of inbreeding depression and founder effects, but also in the accumulation of mutations during maintenance. Males, as are commonly used in gene expression experiments, are expected to display a greater degree of variation between genotypes than females. Our results support this expectation, finding variation in reproductive tissue and an over-representation of gene functions associated with the mitochondria and the defence response. We find further evidence for variability of gene expression in females, a large proportion of which is again attributed to genes with gender-biased expression in reproductive tissue. However, somatic variation is also particularly apparent for female interaction genes that are highly expressed in males.

We note that variation in gene expression between laboratory strains that are apparently the same, the two Oregon R strains from Glasgow and Cambridge, can be substantial. This has implications for genomics studies that combine data from different experiments to make inferences about global processes and our experiments further highlight the importance of controlling genetic background when making inferences from genomic data. Finally, the variation detected in males and females between genotype appears to be largely uncorrelated, and is most likely influenced by alternative underlying mechanisms of divergence. While attempts to determine the forces responsible are beyond the scope of this study, these findings have important implications for the interpretation of gene expression studies that rely on the analysis of a single gender.

## Methods

### Fly Strains

Five long-term laboratory strains of *D. melanogaster *that have been maintained at the University of Cambridge Genetics Department were selected for use in the microarray experiments. These included two strains of "Oregon-R". While Oregon-R was originally collected in 1925 (or earlier) by D. E. Lancefield at Roseburg, this stock has since been maintained at various locations including Glasgow and Cambridge, where stocks were established during the 1970–80s. In addition to the Oregon-R stocks, "wild-type" strains originating from Japan (Hikone), France (Kerbiniou) and the Caribbean (Antigua) were selected. The oldest of these three strains, France (Kerbiniou), was taken in the 1960s. The remain two stocks have been maintained in the laboratory environment for close to two decades.

### Transcriptional Profiling

Three replicate groups of 0–6 hour old males and females were collected for each sex and genotype combination. Total RNA was extracted independently for each of the 30 samples (five lines × two sexes × three replicates) using the TRIzol reagent (GIBCO/BRL). One female replicate of Hikone was later discarded. Samples were treated with DNase and purified on Qiagen RNeasy columns. Biotinylated cRNA probes were hybridized to high-density oligonucleotide Affymetrix *Drosophila *microarrays, as described in the Affymetrix GeneChip Expression Analysis Technical Manual (2000). Array data has been submitted to the Gene Expression Omnibus under the accession numbers GSM231205–GSM231233.

### Statistical Analysis

Data were analysed in the R statistical programming language and development environment, using programs that are maintained as part of the bioconductor suite [65]. To standardize intensity values between arrays, pre-processing of expression values was performed with the robust multi-array analysis package, in which raw intensity values are adjusted via the GC-content of probes in addition to quantile normalization [66,67]. We assessed differential expression of probes with the use of linear modelling and empirical Bayes methods as implemented in the LIMMA package to include the effects of gender, genotype and underlying interactions [26,68]. Multiple testing was accounted for by applying the particularly stringent method of Bonferroni Correction. While we employed a normalization method and multiple correction technique that deals very well with low intensity probe sets, we have also employed a greater set of filters for generating very high confidence data specifically for further analysis of gender by genotype interactions. This additional filter relies on the presence and absence calls of expression provided on Affymetrix arrays with perfect match and mismatch probes. Probes were used in further analysis only if they were deemed to be present in one or more genotype interaction. Finally, probes corresponding to more than one primary FlyBase gene (Mar 2006) were removed from the analysis, while probes corresponding to the same gene were pooled.

Gene Ontology classification was examined for each list of differentially expressed genes (Gene Ontology Constortium, 2001). To identify over-represented GO categories, a hypergeometric test was implemented in R against a subset of common high-level annotations representative of different functions [69]. The resulting genes were also integrated with tissue-specific microarray data from the FlyAtlas database [25]. The presence and absence calls of expression for the testis (excluding accessory glands) and the ovaries (excluding spermatheca and uterus) were used to identify genes expressed in reproductive tissue. Although the data presented here have not been validated by quantitative-PCR, gene expression measures obtained with the Affymetrix platform and quantitative-PCR are know to be highly correlated, if not more conservative in nature [70,71]. The supplementary material for this study are provided in Additional File 1 and 2.

## Authors' contributions

DAB conceived and performed the computational analysis, LM and JW performed the microarray analysis under the supervision of JATD and SR. SR conceived the experiment and raised the funding. JATD and SR raised funding for the Glasgow Affymetrix facility. DAB wrote the paper and all authors read and approved the final manuscript.

## Supplementary Material

Additional file 1 Female by Genotype Supplementary Information. probe_id_affy1: Affymetrix Drosophila 1 probe identifiers. interaction: F × G = female interations; M × G = male interactions. expression: male-biased, female-biased, unbiased gene expression. AveExpr: Average Gene expression in samples. F: The F statistic calculated for Gender by Genotype interaction. adj.P.Val: The adjusted p.value after Bonferroni correction. France_M1 – Hikone_F2: Normalized log intensity values. Female_Only: Indicates whether the gene is expressed only in females. FBID: Flybase gene identifier. CG: DGC gene identifier. GO: Gene Ontology descriptor. probe_id_affy2: Affymetrix Drosophila 2 probe identifiers. BrainCall – MidgutCall: Flyatlas presence calls for tissue expression. tissue: Indicates whether the gene is expressed in either the ovary or somatic tissue Click here for file

Additional file 2 Male by Genotype Supplementary Information. probe_id_affy1: Affymetrix Drosophila 1 probe identifiers. interaction: F × G = female interations; M × G = male interactions. expression: male-biased, female-biased, unbiased gene expression. AveExpr: Average Gene expression in samples. F: The F statistic calculated for Gender by Genotype interaction. adj.P.Val: The adjusted p.value after Bonferroni correction. France_M1 – Hikone_F2: Normalized log intensity values. Male_Only: Indicates whether the gene is expressed only in males. FBID: Flybase gene identifier. CG: DGC gene identifier. GO: Gene Ontology descriptor. probe_id_affy2: Affymetrix Drosophila 2 probe identifiers. BrainCall – MidgutCall: Flyatlas presence calls for tissue expression. tissue: Indicates whether the gene is expressed in either the testes or somatic tissue Click here for file

Additional file 3 Visual representation of Male by Genotype, male-biased and testis specific genes. Relative normalized expression levels for all 5 laboratory strains are displayed with hierarchical clustering. Relative transcriptional levels for genes are represented by green (high) to red (low) colouration. Genes within testis specific expression from EST comparisons are marked in black. Clustering was achieved with euclidean distance on centred gene expression data. Click here for file
