# Supplementary material for: Variable sexually dimorphic gene expression in laboratory strains of Drosophila melanogaster
Source: BMC Genomics. 2007 Dec 10;8:454. doi: 10.1186/1471-2164-8-454 (PMC2244638; doi:10.1186/1471-2164-8-454)
Supplement: Additional file 3 — Visual representation of Male by Genotype, male-biased and testis specific genes. Relative normalized expression levels for all 5 laboratory strains are displayed with hierarchical clustering. Relative transcriptional levels for genes are represented by green (high) to red (low) colouration. Genes within testis specific expression from EST comparisons are marked in black. Clustering was achieved with euclidean distance on centred gene expression data. [file 1471-2164-8-454-S3.pdf]

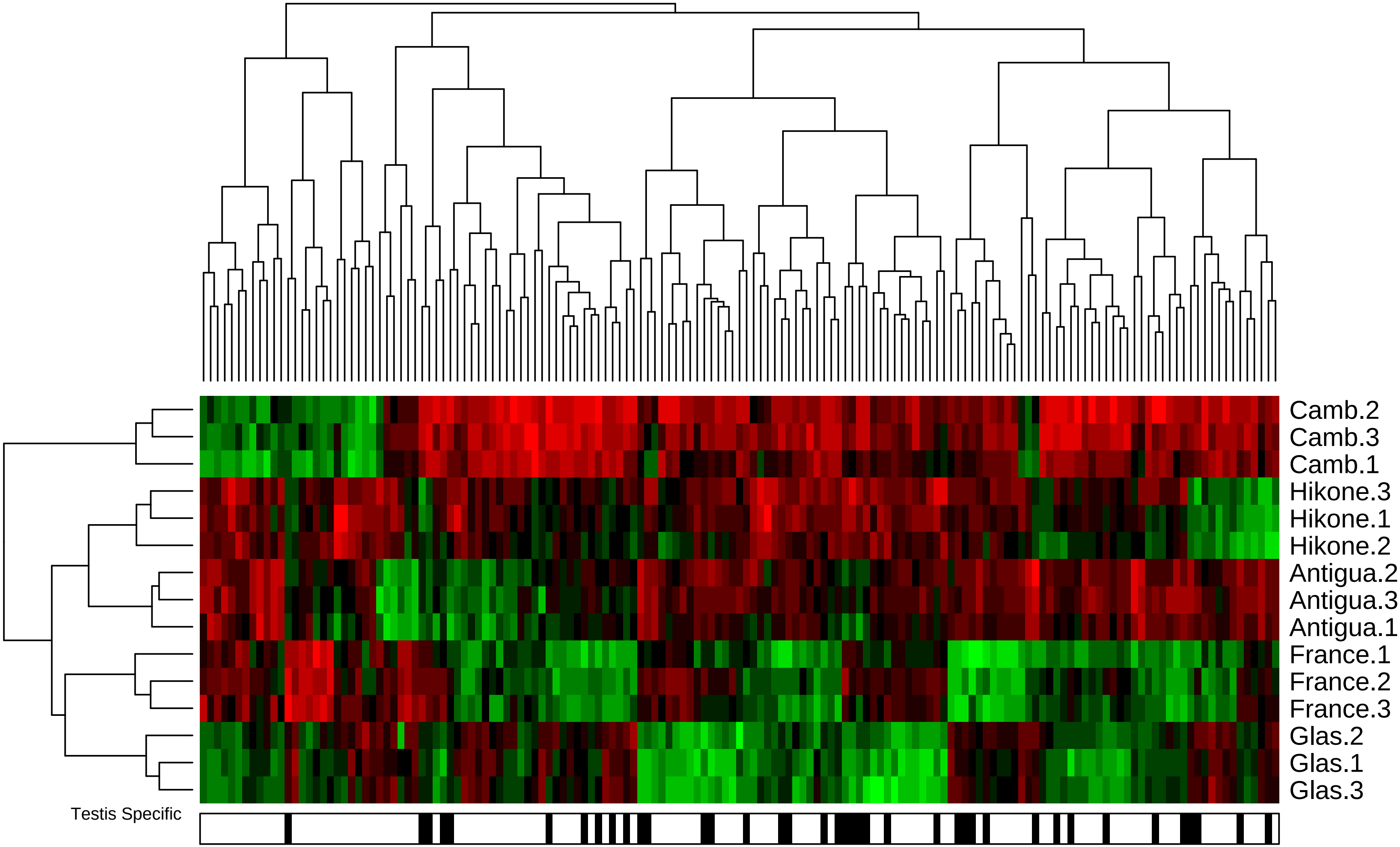
Camb.2
Camb.3
Camb.1
Hikone.3
Hikone.1
Hikone.2
Antigua.2
Antigua.3
Antigua.1
France.1
France.2
France.3
Glas.2
Glas.1
Glas.3
Testis Specific
